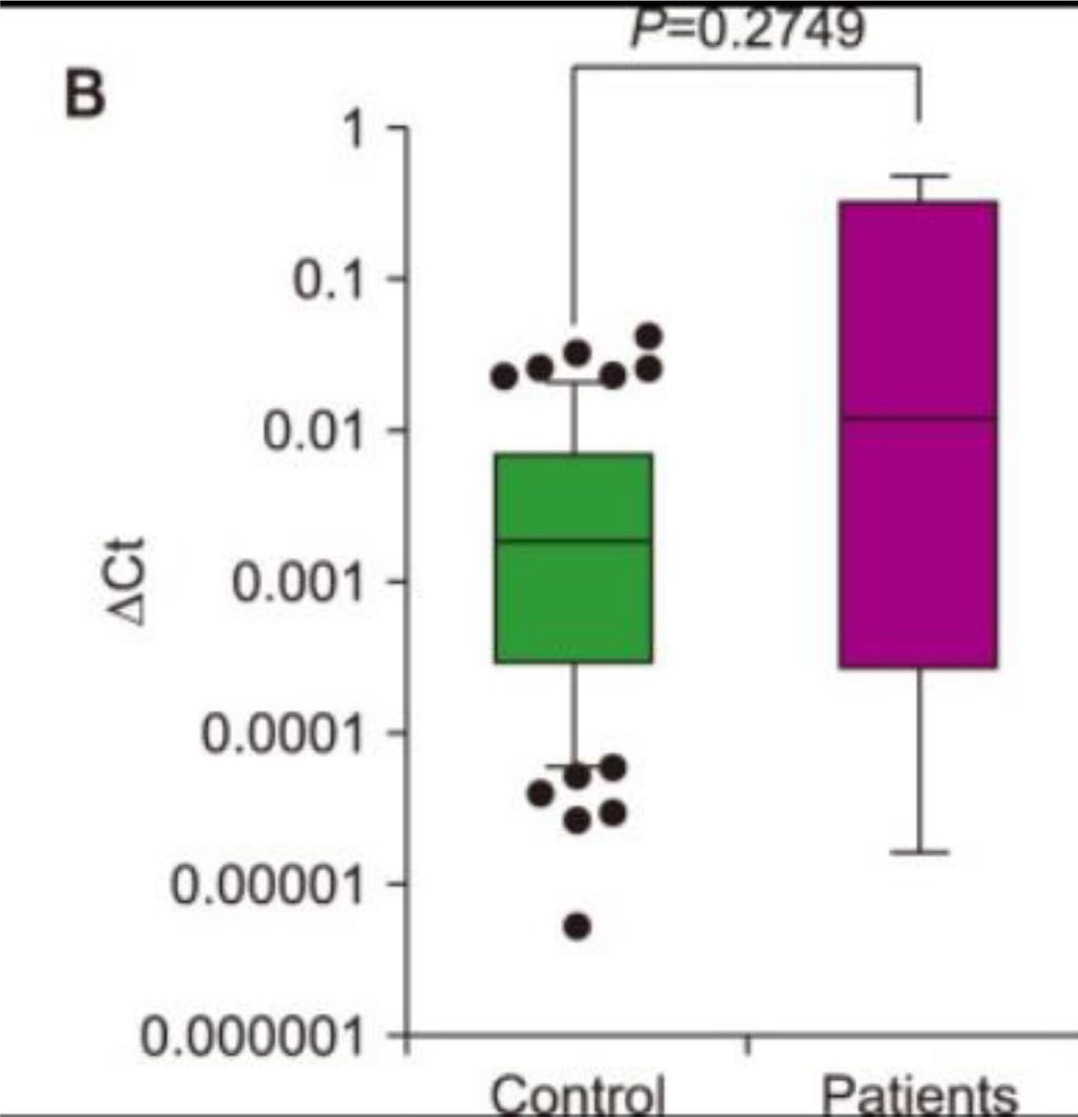# Correction: MicroRNA-765 is upregulated in myelodysplastic syndromes and induces apoptosis via PLP2 inhibition in leukemia cells

**DOI:** 10.1007/s44313-024-00020-y

**Published:** 2024-05-27

**Authors:** Seong-Ho Kang, Ji Seon Choi

**Affiliations:** 1 Department of Laboratory Medicine, Chosun University College of Medicine, Gwangju, Korea; 2 International St. Mary’s Hospital, Catholic Kwandong University, Incheon, Korea

**Correction** **:** **Blood Res 58, 3 (2023)**

**https://doi.org/10.5045/br.2023.2023097**

Following publication of the original article [1], the authors would like to replace Figure 1B.

The correct and incorrect version of Figure 1B are shown below:

Correct
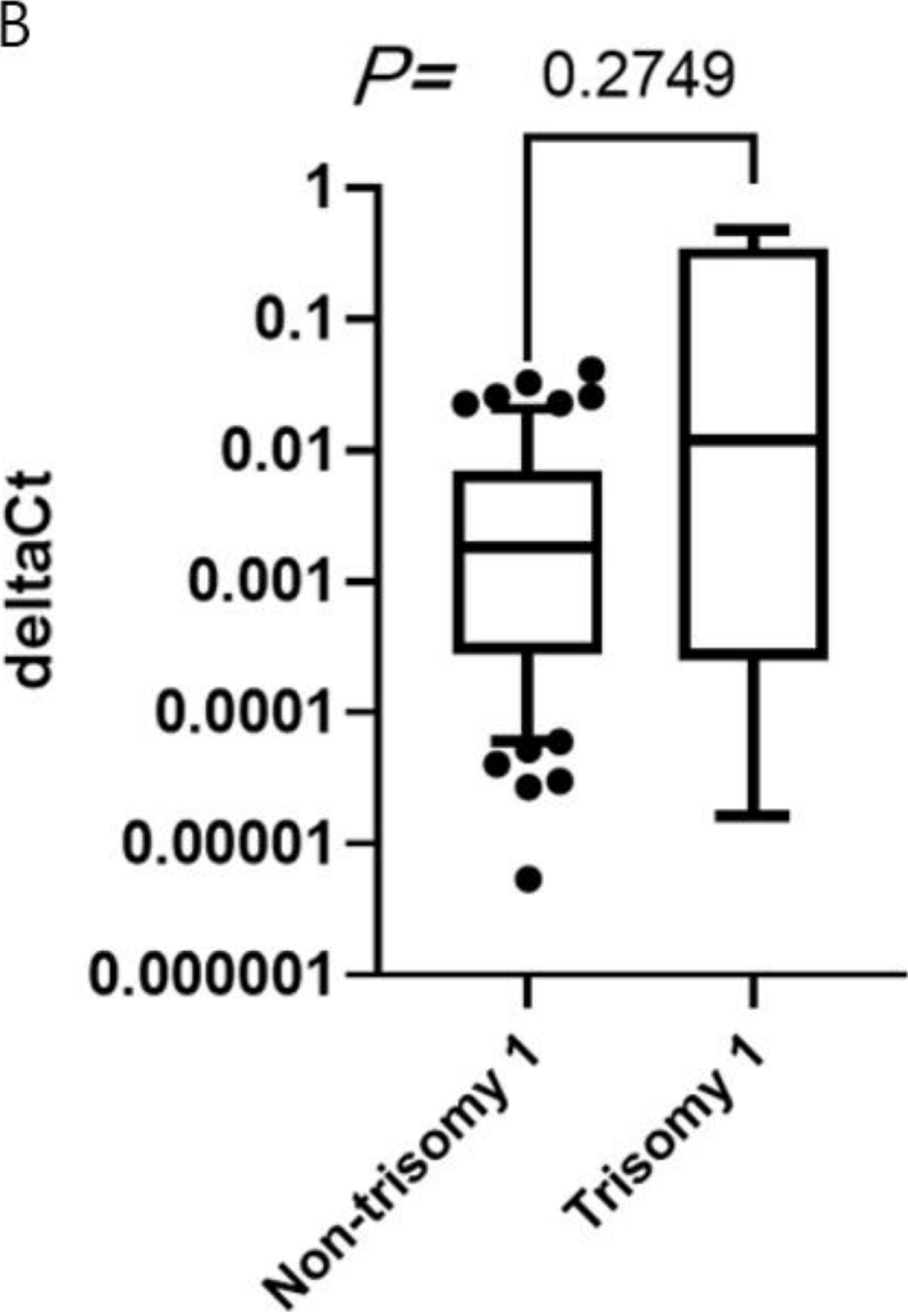


Incorrect